# Predicting Smoking Prevalence in Japan Using Search Volumes in an Internet Search Engine: Infodemiology Study

**DOI:** 10.2196/42619

**Published:** 2022-12-14

**Authors:** Kazuya Taira, Takahiro Itaya, Sumio Fujita

**Affiliations:** 1 Department of Human Health Sciences Graduate School of Medicine Kyoto University Kyoto Japan; 2 Department of Healthcare Epidemiology Graduate School of Medicine and Public Health Kyoto University Kyoto Japan; 3 Yahoo Japan Corporation Tokyo Japan

**Keywords:** health policy, internet use, quality indicators, search engine, smoking, tobacco use, public health, infodemiology, smoking trend, health indicator, health promotion

## Abstract

**Background:**

Tobacco smoking is an important public health issue and a core indicator of public health policy worldwide. However, global pandemics and natural disasters have prevented surveys from being conducted.

**Objective:**

The purpose of this study was to predict smoking prevalence by prefecture and sex in Japan using Internet search trends.

**Methods:**

This study used the infodemiology approach. The outcome variable was smoking prevalence by prefecture, obtained from national surveys. The predictor variables were the search volumes on Yahoo! Japan Search. We collected the search volumes for queries related to terms from the thesaurus of the Japanese medical article database Ichu-shi. Predictor variables were converted to per capita values and standardized as *z* scores. For smoking prevalence, the values for 2016 and 2019 were used, and for search volume, the values for the April 1 to March 31 fiscal year (FY) 1 year prior to the survey (ie, FY 2015 and FY 2018) were used. Partial correlation coefficients, adjusted for data year, were calculated between smoking prevalence and search volume, and a regression analysis using a generalized linear mixed model with random effects was conducted for each prefecture. Several models were tested, including a model that included all search queries, a variable reduction method, and one that excluded cigarette product names. The best model was selected with the Akaike information criterion corrected (AICC) for small sample size and the Bayesian information criterion (BIC). We compared the predicted and actual smoking prevalence in 2016 and 2019 based on the best model and predicted the smoking prevalence in 2022.

**Results:**

The partial correlation coefficients for men showed that 9 search queries had significant correlations with smoking prevalence, including *cigarette* (*r*=*–*0.417, *P*<.001), *cigar* in kanji (*r*=–0.412, *P*<.001), and *cigar* in katakana (*r*=-0.399, *P*<.001). For women, five search queries had significant correlations, including *vape* (*r*=0.335, *P*=.001), *quitting smoking* (*r*=0.288, *P*=.005), and *cigar* (*r*=0.286, *P*=.006). The models with all search queries were the best models for both AICC and BIC scores. Scatter plots of actual and estimated smoking prevalence in 2016 and 2019 confirmed a relatively high degree of agreement. The average estimated smoking prevalence in 2022 in the 47 prefectures for the total sample was 23.492% (95% CI 21.617%-25.367%), showing an increasing trend, with an average of 29.024% (95% CI 27.218%-30.830%) for men and 8.793% (95% CI 7.531%-10.054%) for women.

**Conclusions:**

This study suggests that the search volume of tobacco-related queries in internet search engines can predict smoking prevalence by prefecture and sex in Japan. These findings will enable the development of low-cost, timely, and crisis-resistant health indicators that will enable the evaluation of health measures and contribute to improved public health.

## Introduction

### Smoking Prevalence as a Health Policy Indicator

Tobacco smoking is a cause of many types of cancer, respiratory disease, and coronary artery disease [1-4]. Since 2013, various electronic nicotine delivery systems (ENDS) and heat-not-burn tobacco products have been introduced as alternatives to cigarettes [5], with 3 brands available in Japan: Iqos, Glo, and Ploom Tech. These ENDS and heat-not-burn tobacco products have been reported to be potentially harmful and alerts have been issued [6,7].

Smoking prevalence is included as an indicator in the Health Japan 21 (the Second Term), a guideline for health measures in Japan, and policy measures are being implemented based on target values [8]. Currently, smoking prevalence is ascertained through national surveys, such as the Comprehensive Survey of Living Conditions [9] and the National Health and Nutrition Survey [10]. The Comprehensive Survey of Living Conditions is conducted every 3 years, and the National Health and Nutrition Survey is conducted every 5 years on a large scale. There are only a limited number of surveys, which require enormous effort and cost in the millions of dollars, that have a large enough sample size to be tabulated by prefecture. Furthermore, these surveys were not conducted in years with major natural disasters, such as the 2016 Kumamoto earthquake; moreover, after 2020, due to the COVID-19 pandemic, the Comprehensive Survey of Living Conditions in 2020 and the National Health and Nutrition Survey in 2020 and 2021 were cancelled. The discontinuation of these large-scale national surveys monitoring health indicators has prevented the evaluation of policies and hindered evidence-based policy making.

### Trends in Smoking Prevalence in Japan

Smoking prevalence in Japan has decreased dramatically, from 82.3% in 1965 to 27.8% in 2018 for men over 20 years old and from 16.5% in 1965 to 8.7% in 2018 for women [11]. However, Japan has a slightly higher smoking prevalence than other high-income countries, indicating that tobacco control measures have not yet reached the level of best practice [12]. In addition, it has been pointed out that socioeconomic disparities may be a factor associated with Japan’s persistent decline in smoking prevalence [13], which is an important public health issue.

### Internet Search Engine and Tobacco Use

Significant correlations have been reported between internet search trends and the prevalence of tobacco and smokeless tobacco use by state in the United States [14]. Moreover, youth are consistently exposed to tobacco-related content on the internet [15], and exposure to tobacco-related content on social media has been reported to be a risk for smoking behavior [16]. Therefore, we hypothesized that it would be possible to predict smoking prevalence at the regional level based on search trends on the internet. In addition, it has been reported that internet search trends can track users’ interest in ENDS [17] and heat-not-burn tobacco [18]. Thus, the purpose of this study was to predict smoking prevalence by prefecture and sex in Japan based on internet search trends.

## Methods

This study used the infodemiology approach to monitoring smoking prevalence in Japan based on internet search engine trends.

### Outcome Variable

The outcome variable was smoking prevalence. Smoking prevalence was obtained for each prefecture from the Comprehensive Survey of Living Conditions [9], a national survey conducted by the Japanese government. This is a national survey of households that are randomly selected and stratified by region from all over Japan. A simple survey (distributed to approximately 55,000 households, comprising 138,000 people) is conducted annually, and a large-scale survey (distributed to approximately 277,000 households, comprising 688,000 people) is conducted every 3 years. Because smoking prevalence is included only in the larger-scale survey, we obtained smoking prevalence for each prefecture in 3-year periods from 2001 to 2019 (Multimedia Appendix 1). However, in 2016, data from Kumamoto prefecture were missing due to a natural disaster caused by an earthquake. Since the volume of data was too small to impute missing values, and they could not be properly estimated, complete case analyses were conducted in this study.

### Predictors

The predictor variable was search volumes on Yahoo! Japan Search, one of the largest search engines in Japan. This study was conducted in collaboration with Yahoo Japan Corporation, and the researchers were authorized to access search log data from Yahoo! Japan Search. The necessary data were extracted with a tabulation program that accessed the Yahoo Japan Corporation server via a virtual private network connection. The search queries for which search volumes were extracted were words that were listed as synonyms for “cigarette,” “smoking,” and “e-cigarette” in the thesaurus of Ichu-shi Web, the largest Japanese medical article search database. The monthly number of searches per prefecture for each search query was obtained, and the total number of searches per fiscal year (FY, running from April 1 to March 31) was calculated.

Because the Yahoo! Japan search log data were available starting for the year 2014, to predict smoking prevalence by search volume in the FYs before the FY in which we wanted to predict smoking prevalence, we obtained search volumes for FYs 2015, 2018, and 2021. These were all years during which the triennial large-scale survey of smoking prevalence was conducted, making retrospective data available. The reason for predicting smoking prevalence in FYs is that Japanese local governments evaluate their projects every FY. Queries that were not written in Japanese or that had a month in which they were never searched for were excluded; 18 queries were thus used in the analysis (Textbox 1).

**Search queries used in the analysis, with original Japanese-language terms. The multiple entries for some terms reflect the multiple Japanese writing systems, including hiragana, katakana, kanji, and the Latin alphabet.**

**Search terms (with Japanese text)**

- *Cigarette* in katakana (シガレット)
- *Tobacco* in hiragana (たばこ), *tobacco* in katakana (タバコ), *tobacco* in kanji (煙草)
- *Cigar* in kanji (葉巻), *cigar* in katakana (シガー)
- *Glo* (glo), *glo* in katakana (グロー)
- *Vape* (vape)
- *Iqos* (iqos), *iqos* in katakana (アイコス)
- *Ploom Tech* in katakana (プルームテック)
- *Electronic cigarette* in katakana (電子タバコ), *electronic cigarette* in hiragana (電子たばこ)
- *Heat-not-burn tobacco* in hiragana (加熱式たばこ), *heat-not-burn tobacco* in katakana (加熱式タバコ)
- *Smoking* in kanji (喫煙)
- *Quitting smoking* in kanji (禁煙)

### Standardization of Predictors

Since the male to female ratio for each search query can be obtained based on registration information from user IDs, search volumes by sex were calculated by prorating the search volumes by sex. Since search volumes are affected by the size of the prefectural population, the search volumes per capita were calculated by dividing the total or male/female prefectural population and then converting the results to a *z* score to standardize the results. The *z* scores were calculated with the following formula (“query A” refers to each search query used in the analysis):




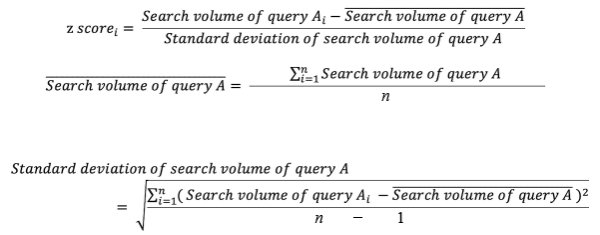




The value for the prefectural populations used to calculate the search volumes per capita was the value on October 1 (the median day of the FY) of the respective year, obtained from open government data on population estimate statistics [19].

### Statistical Analysis

Partial Pearson correlation coefficients, adjusted for data year, between smoking prevalence per prefecture and the *z* scores of the search volumes for each query for each prefecture were calculated. Regression analysis using a generalized linear mixed model (GLMM) was conducted with smoking prevalence as the outcome variable, *z* scores for each search query and survey year as predictor variables, and random effects for each prefecture. For the regression analysis, we used data from 2016 and 2019 for both smoking prevalence and number of searches. In the regression analyses, model 0 included only the survey year and intercept as predictors; model 1 included the survey year, intercept, and queries, but excluded the names of tobacco-related products; model 2 included the survey year, intercept, and queries selected by the backward selection method; and model 3 included the survey year, intercept, and all search queries in the total sample for men and women separately. The selection of the best model was determined using the Akaike information criterion corrected (AICC) for small sample size and the Bayesian information criterion (BIC). The AICC and BIC are model fit indices that focus on prediction accuracy, and both refer to better fit with smaller values relative to other models. The analyses were conducted using SPSS Statistics (version 27; IBM). The significance level was set at 1% for all analyses.

### Evaluation of the GLMM Model and Prediction of Smoking Prevalence in 2022

Search volumes were substituted into the selected best model to predict smoking prevalence for 2016, 2019, and 2022. For 2016 and 2019, scatter plots of actual and estimated smoking prevalence were drawn to confirm agreement. The smoking prevalence in 2022 was plotted as a line graph along with actual values from 2001 to 2019 to confirm the trend.

### Ethical Considerations

This study involved secondary analysis of public statistics and anonymized existing data; therefore, ethical review was waived by the Kyoto University Graduate School and Faculty of Medicine Ethics Committee.

## Results

### Correlation Coefficients Between Smoking Prevalence and Each Search Query

The results of the partial correlation analyses are presented in Table 1. For men, the following 9 search queries had significant correlations with smoking prevalence: *cigar* in katakana (シガー; *r*=–0.399, *P*<.001), *cigarette* in katakana (シガレット; *r*=–0.417, *P*<.001), *tobacco* in katakana (タバコ; *r*=–0.388, *P*<.001), *tobacco* in hiragana (たばこ; *r*=–0.334, *P*=.001), *tobacco* in kanji (煙草; *r*=–0.370, *P*<.001), *smoking* in kanji (喫煙; *r*=–0.346, *P*=.001), *electronic cigarette* in katakana (電子タバコ; *r*=–0.303, *P*=.003), *electronic cigarette* in hiragana (電子たばこ; *r*=–0.271, *P*=.009), and *cigar* in kanji (葉巻; *r*=–0.412, *P*<.001). For women, the following 5 search queries had significant correlations: *vape*, (*r*=0.335, *P*=.001), *cigar* in katakana (シガー; *r*=0.286, *P*=.006), *quitting smoking* in kanji (禁煙; *r*=0.288, *P*=.005), *electronic cigarette* in katakana (電子タバコ; *r*=0.271, *P*=.009), and *electronic cigarette* in hiragana (電子たばこ; *r*=0.271, *P*=.009) (Table 1).

**Table 1:** Partial Pearson correlation coefficient (r) between prefectural smoking prevalence and the search volumes of each query by sex.

Search term	Total			Men			Women	
	*r*	*P* value	*r*		*P* value	*r*		*P* value
*Glo*	0.02	*P*=.89	–0.16		*P*=.14	0.22		*P*=.04
*Iqos*	–0.01	*P*=.96	–0.20		*P*=.06	0.18		*P*=.08
*Vape*	0.01	*P*=.89	–0.25		*P*=.02	0.34		*P*=.001
*Iqos* (アイコス)	0.01	*P*=.92	–0.21		*P*=.04	0.26		*P*=.01
*Glo* (グロー)	0.03	*P*=.77	–0.09		*P*=.39	0.19		*P*=.07
*Cigar* (シガー)	–0.12	*P*=.25	–0.40		*P*<.001	0.29		*P*=.006
*Cigarette* (シガレット)	–0.20	*P*=.06	–0.42		*P*<.001	0.18		*P*=.09
*Tobacco* (タバコ)	–0.11	*P*=.31	–0.39		*P*<.001	0.25		*P*=.02
*Tobacco* (たばこ)	–0.10	*P*=.36	–0.33		*P*=.001	0.23		*P*=.03
*Ploomtech* (プルームテック)	0.001	*P*=.99	–0.16		*P*=.14	0.20		*P*=.06
*Tobacco* (煙草)	–0.11	*P*=.30	–0.37		*P*<.001	0.24		*P*=.02
*Heat-not-burn tobacco* (加熱式タバコ)	–0.07	*P*=.46	–0.23		*P*=.03	0.16		*P*=.13
*Heat-not-burn tobacco* (加熱式たばこ)	0.01	*P*=.93	–0.15		*P*=.14	0.20		*P*=.06
Smoking (喫煙)	–0.11	*P*=.31	–0.35		*P*=.001	0.19		*P*=.07
Quitting smoking (禁煙)	0.02	*P*=.85	–0.21		*P*=.05	0.29		*P*=.005
Electronic cigarette (電子タバコ)	–0.05	*P*=.67	–0.30		*P*=.003	0.27		*P*=.009
Electronic cigarette (電子たばこ)	–0.03	*P*=.79	–0.27		*P*=.009	0.27		*P*=.009
Cigar (葉巻)	–0.15	*P*=.16	–0.41		*P*<.001	0.21		*P*=.046

### Results of the Regression Analyses and Evaluation of the Best Models

**Figure 1:**
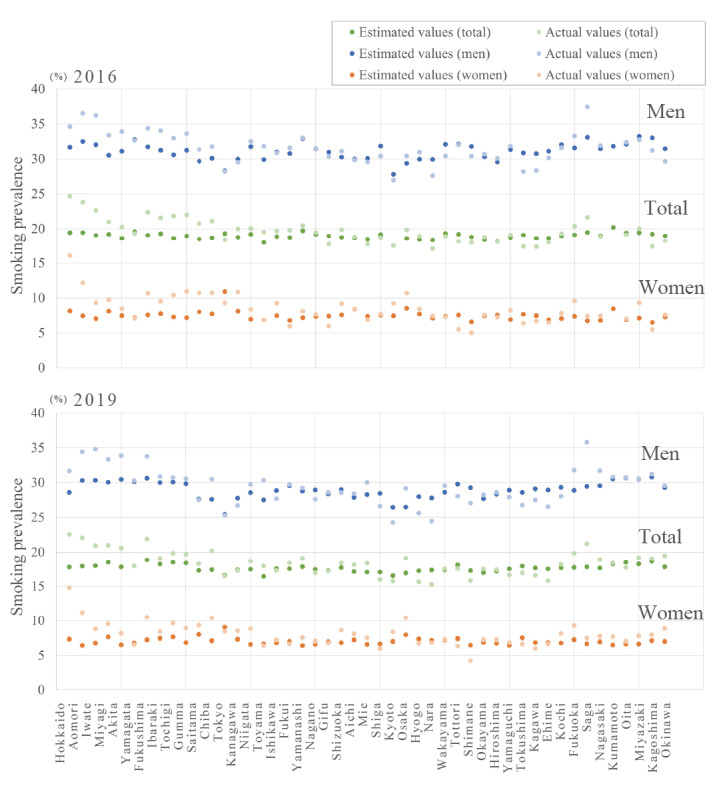
Comparison of actual and modeled estimates of smoking prevalence by prefecture in 2016 and 2019.

Model 3 was the best model for both AICC and BIC scores in all regression analyses for the total sample (AICC 308.043 and BIC 312.452), men (AICC 338.656 and BIC 343.066), and women (AICC 302.225 and BIC 306.635). Details are provided in Tables S1, S2, and S3 in Multimedia Appendix 2. Moreover, no search queries with significant regression coefficients were found in model 3 for either sex or the total sample. Scatter plots of actual and estimated smoking prevalence in 2016 and 2019 confirmed a relatively high degree of agreement (Figure 1).

### Estimates of Smoking Prevalence by Prefecture in 2022

**Figure 2:**
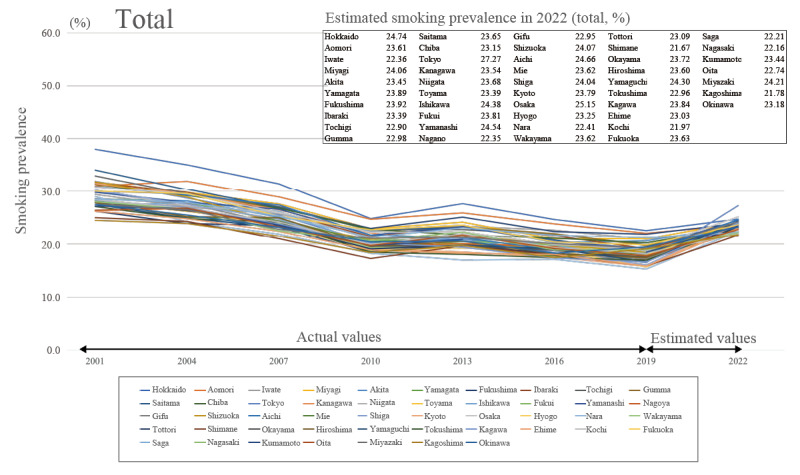
Total trends in smoking prevalence and predicted smoking prevalence in 2022.

**Figure 3:**
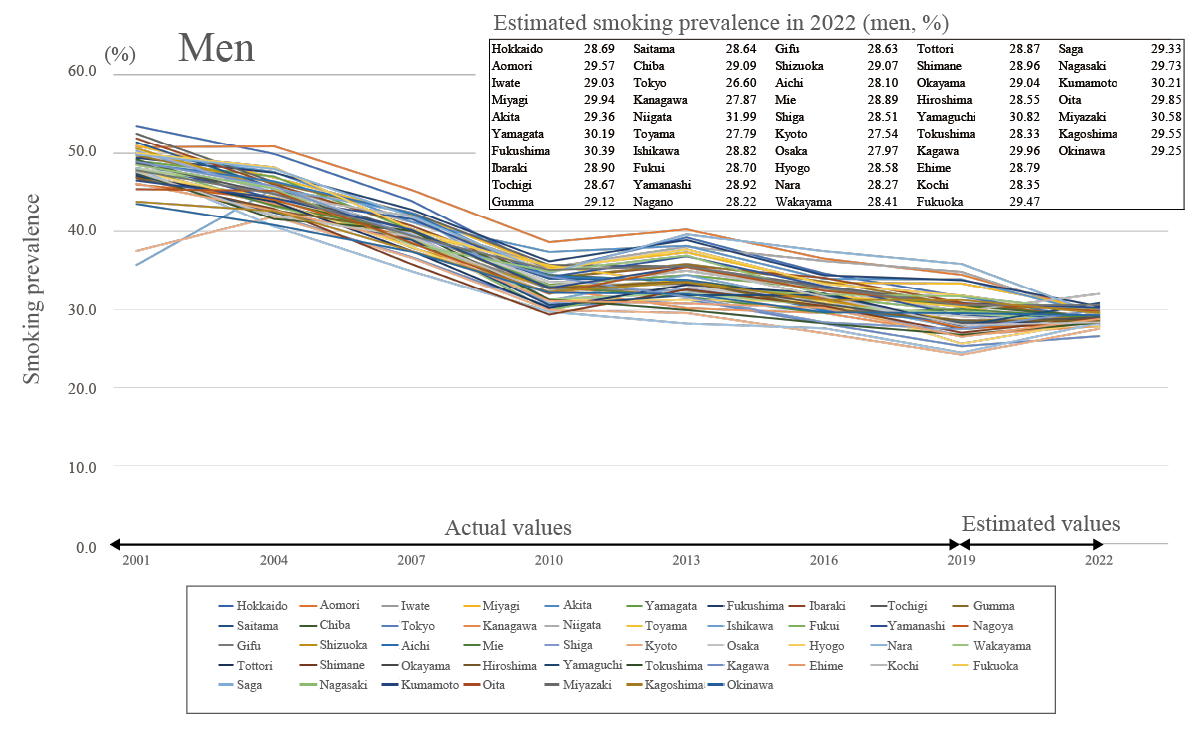
Trends in smoking prevalence and predicted smoking prevalence in 2022 for men.

**Figure 4:**
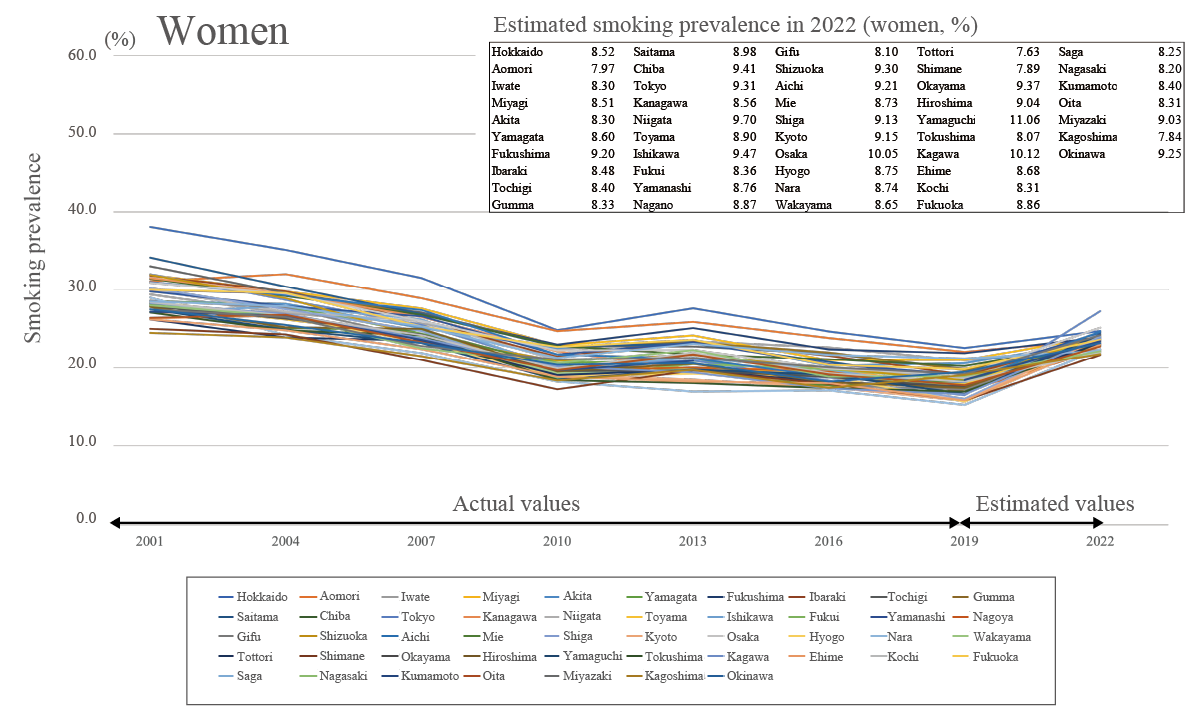
Trends in smoking prevalence and predicted smoking prevalence in 2022 for women.

The average estimated smoking prevalence in 2022 for the 47 prefectures for the total sample, including men and women (Figure 2), was 23.492% (95% CI 21.617%-25.367%), showing an increasing trend, with a prevalence of 29.024% (95% CI 27.218%-30.830%) for men (Figure 3) and 8.793% (95% CI 7.531%-10.054%) for women (Figure 4).

## Discussion

### Principal Results

We found that smoking prevalence could be predicted with a moderate degree of accuracy using a GLMM based on search volume for tobacco-related queries in internet search engines. A univariate analysis of the partial correlation coefficients between smoking prevalence by prefecture and each search query revealed significant variables only for sex, but the GLMM predictions had approximately the same predictive accuracy for the total sample as for men and women. The GLMM models were judged from the AICC and BIC scores, and the models that included all search queries as predictors were adopted as the best models for all sexes and the total sample. These results suggest that rather than specific words being strong predictors of smoking prevalence, it is more likely that different words play a role in revealing prefectural-specific characteristics of monotonic smoking prevalence trends.

When actual and estimated smoking prevalence were compared, the estimated values tended to show less variation, and this may reduce the accuracy of predicting prefectures with smoking prevalence that deviates from the mean. However, this allows us to follow trends in smoking prevalence for Japan overall. Smoking prevalence has continued to show a consistent decreasing trend from 2001 to 2019, but estimates for 2022 show a flat trend or an increasing trend among women. After 2020, the aftereffects of the COVID-19 pandemic have caused behavioral restrictions and stagnating economic activity around the world, and Japan is in the midst of the seventh wave as of 2022, with the highest number of cases in the world [20]. This could be one contributor to the trends seen in this study. Economic disparity has also been identified as a contributing factor to the still-high prevalence of smoking in Japan [13]. Therefore, economic deprivation attributed to COVID-19 may be related to the increase in the estimated smoking prevalence in 2022.

More interestingly, the correlation coefficients between the search volume of tobacco-related queries and smoking prevalence were negative for men, positive for women, and uncorrelated in the total sample. Smoking prevalence in Japan has traditionally differed significantly between men and women, which could lead to relatively higher search volumes for smoking cessation behaviors in men and higher search volumes for smoking behaviors in women. Notably, the COVID-19 pandemic has disproportionately affected women’s employment, a phenomenon called the “she-cession” [21]. Several studies have also reported an increase in suicide and mental health problems among women in Japan during the COVID-19 pandemic [22,23]. Therefore, it is possible that smoking prevalence among women increased as a coping behavior for stress [24].

Hence, when considering health policies based on the search volume of tobacco-related queries in internet search engines, men and women require different approaches. It would be desirable for future studies to not only predict smoking prevalence, but also investigate smokers’ search behavior patterns and gender differences in more detail, clarifying the relationship between smokers’ actual search behavior and smoking prevalence.

### Limitations

One of the limitations of our study relates to the flexibility of the model, as both smoking prevalence and tobacco-related query retrieval volume statistics were only available for 2 years (2016 and 2019). In particular, for Kumamoto prefecture, data were missing due to a major earthquake in 2016, so care must be taken in interpreting the estimates. The lack of statistically significant variables in the GLMM regression analysis may also be due to the small sample size and multicollinearity in the highly correlated relationships among search volumes for the tobacco-related queries. This study did not address this issue, because it focused on the accuracy of predicting smoking prevalence as an outcome. However, if these limitations can be resolved in future analyses using more sophisticated statistical methods, we could obtain insights into factors that influence changes in smoking prevalence. In addition, internet usage in Japan differs by age group: more than 90% of people between their teenage years and the sixth decade of life use the internet, while 74.2% of those in their sixties and 57.5% of those in their eighties use the internet [25], suggesting that the influence of the elderly may have been underestimated. However, as of 2019, the highest smoking prevalence in men was among those in their forties, at 36.5%, decreasing as people reached their sixties; the prevalence was 31.1% among those in their sixties and 15.1% among those aged 70 or older. The same trend was observed in women, with the highest smoking prevalence being 12.9% among those in their fifties, compared to 8.6% of those in their sixties and 3% in women aged 70 and older. Therefore, we believe that the impact of lower internet use among the elderly was limited. Finally, although this study used separate analyses for men and women, it is possible that gender bias was present in the internet search behavior. One previous study in Australia [26] suggests that female smokers who are highly socially disadvantaged seem to use the internet more frequently; however, there is no evidence of gender differences in internet search behavior in the general population in Japan. Research on gender bias in smokers’ internet search behavior is also an issue for future study.

### Comparison With Prior Work

Most previous studies in the field of infoveillance have focused on predicting the prevalence of infectious diseases, such as influenza [27,28]. This study suggests that smoking prevalence can also be predicted with high accuracy by the search volume of tobacco-related queries. Many previous studies using search engines have used Google Trends data, which does not provide search volume by sex or prefecture, unlike the data from Yahoo searches, making the latter more appropriate to conduct our research. Although we found no studies that predicted smoking prevalence by internet search volume, a moderate correlation between smoking prevalence and search volumes for tobacco-related queries was reported in the United States [14]. In this study, the same trend was observed for Japanese men, with a slightly weaker correlation for Japanese women. The US smoking prevalence in 2020 was 14.1% for men and 11% for women [29], with no significant difference. However, Japan has a very large gap in smoking prevalence between men and women, which may be the reason for the slightly weaker correlation for women.

Our findings may be useful in the evaluation of public health measures when large-scale nationwide surveys are not possible due to epidemics or natural disasters such as major earthquakes. In fact, Japan has a history of missing statistics, including statistics related to COVID-19 and the Kumamoto earthquake, and has a high probability of large-scale earthquakes and volcanic eruptions in the future.

### Conclusions

This study suggests that internet search volume for tobacco-related queries can predict smoking prevalence by prefecture. Our findings may facilitate the development of low-cost, timely, and crisis-resistant health indicators that will enable the evaluation of health measures and contribute to improved public health.

## Appendix

Multimedia Appendix 1 Smoking prevalence by prefecture, 2001-2019.

Multimedia Appendix 2 Generalized linear mixed models for both sexes, men, and women.

## Abbreviations

AICC: Akaike information criterion corrected for small sample size
BIC: Bayesian information criterion
ENDS: electronic nicotine delivery system
FY: fiscal year
GLMM: generalized linear mixed model
