# Supplementary material for: Predicting Smoking Prevalence in Japan Using Search Volumes in an Internet Search Engine: Infodemiology Study
Source: J Med Internet Res. 2022 Dec 14;24(12):e42619. doi: 10.2196/42619 (PMC9798260; doi:10.2196/42619)
Supplement: Multimedia Appendix 1 [file jmir_v24i12e42619_app1.docx]

## Multimedia Appendix

Appendix 1. Smoking prevalence by prefecture, 2001-2019

|  | 2001 | | | 2004 | | | 2007 | | | 2010 | | | 2013 | | | 2016 | | | 2019 | | |
| --- | --- | --- | --- | --- | --- | --- | --- | --- | --- | --- | --- | --- | --- | --- | --- | --- | --- | --- | --- | --- | --- |
|  | Total | Men | Wo  men | Total | Men | Wo  men | Total | Men | Wo  men | Total | Men | Wo  men | Total | Men | Wo  men | Total | Men | Wo  men | Total | Men | Wo  men |
| Hokkaido | 38.01 | 53.50 | 24.34 | 35.12 | 49.93 | 22.23 | 31.47 | 43.85 | 20.65 | 24.84 | 35.01 | 16.15 | 27.63 | 39.25 | 17.84 | 24.66 | 34.62 | 16.08 | 22.57 | 31.65 | 14.82 |
| Aomori | 31.05 | 50.76 | 14.10 | 31.97 | 50.88 | 15.41 | 28.91 | 45.28 | 14.72 | 24.72 | 38.63 | 12.70 | 25.90 | 40.25 | 14.34 | 23.84 | 36.51 | 12.16 | 22.06 | 34.44 | 11.19 |
| Iwate | 29.42 | 49.80 | 11.39 | 27.01 | 45.56 | 10.28 | 25.69 | 41.25 | 10.18 | 22.40 | 35.39 | 10.11 | 23.40 | 38.03 | 10.22 | 22.63 | 36.22 | 9.31 | 20.94 | 34.82 | 8.78 |
| Miyagi | 31.92 | 51.04 | 14.36 | 29.83 | 46.88 | 14.38 | 27.63 | 42.28 | 14.29 | 22.93 | 35.37 | 11.19 | 24.15 | 37.33 | 11.92 | 20.99 | 33.41 | 9.74 | 21.01 | 33.33 | 9.52 |
| Akita | 28.53 | 49.88 | 10.08 | 28.15 | 47.57 | 10.88 | 25.11 | 41.19 | 11.06 | 22.54 | 37.38 | 9.78 | 23.54 | 38.17 | 10.58 | 20.26 | 33.93 | 8.48 | 20.60 | 33.87 | 8.18 |
| Yamagata | 28.62 | 49.02 | 10.71 | 27.94 | 46.95 | 10.79 | 24.43 | 40.18 | 9.86 | 20.64 | 33.18 | 8.86 | 20.88 | 34.43 | 9.11 | 19.30 | 32.63 | 7.11 | 17.97 | 30.27 | 6.59 |
| Fukushima | 30.03 | 49.42 | 12.57 | 29.53 | 47.54 | 12.76 | 26.88 | 42.82 | 12.15 | 22.99 | 36.17 | 10.53 | 25.11 | 38.88 | 12.06 | 22.39 | 34.38 | 10.68 | 21.90 | 33.77 | 10.50 |
| Ibaraki | 31.81 | 51.93 | 12.80 | 29.77 | 46.02 | 13.57 | 26.01 | 40.75 | 11.82 | 21.45 | 34.05 | 9.08 | 23.33 | 35.64 | 11.62 | 21.58 | 34.06 | 9.54 | 19.08 | 30.86 | 8.40 |
| Tochigi | 32.96 | 52.46 | 15.04 | 29.64 | 46.34 | 13.91 | 27.10 | 42.44 | 12.66 | 22.77 | 35.70 | 10.69 | 22.74 | 35.54 | 11.18 | 21.84 | 32.98 | 10.42 | 19.85 | 30.72 | 9.62 |
| Gumma | 31.39 | 49.66 | 14.59 | 29.41 | 46.25 | 13.50 | 26.94 | 42.19 | 12.37 | 22.76 | 34.05 | 11.25 | 23.29 | 35.83 | 10.45 | 21.99 | 33.60 | 10.94 | 19.68 | 30.54 | 8.94 |
| Saitama | 34.06 | 51.45 | 16.98 | 30.43 | 45.55 | 15.55 | 26.91 | 39.95 | 14.37 | 22.73 | 33.95 | 11.84 | 23.13 | 33.81 | 13.12 | 20.76 | 31.36 | 10.72 | 18.29 | 27.52 | 9.34 |
| Chiba | 31.40 | 49.15 | 14.05 | 29.19 | 43.97 | 15.11 | 26.81 | 39.71 | 13.98 | 22.81 | 34.44 | 11.55 | 21.75 | 33.56 | 10.67 | 21.11 | 31.75 | 10.75 | 20.22 | 30.48 | 10.42 |
| Tokyo | 32.03 | 47.21 | 17.40 | 28.69 | 42.03 | 16.02 | 25.49 | 36.70 | 14.86 | 20.29 | 30.34 | 11.36 | 20.93 | 31.26 | 11.54 | 18.33 | 28.20 | 9.32 | 16.50 | 25.34 | 8.44 |
| Kanagawa | 31.50 | 47.18 | 16.32 | 29.53 | 43.50 | 15.72 | 26.46 | 38.47 | 14.72 | 22.13 | 32.69 | 11.86 | 19.75 | 30.15 | 9.75 | 19.98 | 29.54 | 10.85 | 17.29 | 26.73 | 8.53 |
| Niigata | 29.95 | 50.70 | 10.51 | 27.20 | 44.67 | 11.55 | 26.04 | 41.90 | 11.37 | 21.00 | 33.00 | 9.71 | 21.66 | 34.95 | 9.14 | 20.04 | 32.49 | 8.39 | 18.69 | 29.75 | 8.82 |
| Toyama | 28.02 | 47.00 | 10.78 | 26.17 | 44.42 | 9.76 | 23.93 | 39.71 | 9.61 | 20.42 | 35.64 | 7.46 | 19.70 | 33.25 | 7.96 | 19.52 | 31.82 | 6.86 | 17.99 | 30.33 | 6.44 |
| Ishikawa | 27.05 | 35.67 | 18.80 | 27.93 | 45.52 | 12.10 | 26.21 | 42.38 | 12.53 | 19.82 | 31.12 | 9.29 | 21.33 | 34.43 | 8.98 | 19.69 | 30.88 | 9.26 | 17.32 | 27.67 | 7.22 |
| Fukui | 28.34 | 48.81 | 9.45 | 26.53 | 43.23 | 9.69 | 24.31 | 39.19 | 8.07 | 18.70 | 29.90 | 6.15 | 20.46 | 33.33 | 7.26 | 19.80 | 31.60 | 5.97 | 18.42 | 29.73 | 6.69 |
| Yamanashi | 29.80 | 48.72 | 12.03 | 27.88 | 44.24 | 12.65 | 26.60 | 41.64 | 12.65 | 21.72 | 34.18 | 9.30 | 23.30 | 36.81 | 10.98 | 20.46 | 33.02 | 8.11 | 19.13 | 29.21 | 7.55 |
| Nagano | 27.21 | 46.00 | 9.75 | 26.48 | 44.14 | 10.27 | 23.65 | 38.99 | 8.70 | 19.66 | 32.74 | 8.22 | 20.02 | 31.86 | 8.70 | 19.49 | 31.53 | 7.66 | 16.97 | 27.60 | 7.07 |
| Gifu | 28.56 | 47.98 | 10.71 | 27.71 | 45.75 | 11.07 | 23.66 | 39.07 | 9.62 | 19.53 | 32.63 | 7.54 | 20.52 | 32.41 | 9.65 | 17.75 | 30.35 | 5.99 | 17.23 | 28.57 | 6.81 |
| Shizuoka | 31.90 | 50.60 | 14.32 | 28.91 | 45.54 | 13.64 | 24.00 | 37.93 | 10.75 | 20.91 | 32.91 | 9.69 | 21.67 | 33.62 | 10.61 | 19.90 | 31.12 | 9.19 | 18.42 | 28.55 | 8.62 |
| Aichi | 31.04 | 48.84 | 13.01 | 29.29 | 46.39 | 13.05 | 27.38 | 42.33 | 12.75 | 21.72 | 34.26 | 9.86 | 21.19 | 33.75 | 9.35 | 18.83 | 29.84 | 8.47 | 18.14 | 28.41 | 8.09 |
| Mie | 28.16 | 48.02 | 10.81 | 25.11 | 43.31 | 9.27 | 24.77 | 39.85 | 10.20 | 20.27 | 32.94 | 9.14 | 19.36 | 31.59 | 7.89 | 17.71 | 29.55 | 6.90 | 18.37 | 30.03 | 7.53 |
| Shiga | 30.17 | 50.31 | 11.31 | 27.50 | 44.99 | 11.28 | 24.71 | 39.64 | 9.18 | 19.13 | 30.75 | 7.49 | 19.81 | 32.69 | 7.71 | 18.72 | 30.39 | 7.71 | 16.02 | 26.63 | 6.00 |
| Kyoto | 26.35 | 37.54 | 16.40 | 26.69 | 41.90 | 13.53 | 23.97 | 36.62 | 12.69 | 19.10 | 29.93 | 9.73 | 18.49 | 29.55 | 8.47 | 17.51 | 26.96 | 9.23 | 15.78 | 24.26 | 8.36 |
| Osaka | 30.95 | 48.06 | 15.69 | 29.52 | 45.69 | 15.19 | 26.05 | 39.77 | 13.78 | 22.26 | 33.56 | 12.28 | 22.33 | 33.05 | 12.89 | 19.87 | 30.44 | 10.71 | 19.15 | 29.14 | 10.42 |
| Hyogo | 28.50 | 47.72 | 11.51 | 26.52 | 43.77 | 11.53 | 23.26 | 37.99 | 10.51 | 18.97 | 31.25 | 8.17 | 19.21 | 31.20 | 8.72 | 18.90 | 30.98 | 8.42 | 15.66 | 25.65 | 6.78 |
| Nara | 29.00 | 48.11 | 11.61 | 24.23 | 40.64 | 9.33 | 21.90 | 34.91 | 9.50 | 18.22 | 29.72 | 7.83 | 16.96 | 28.21 | 7.16 | 17.10 | 27.60 | 7.44 | 15.28 | 24.49 | 6.89 |
| Wakayama | 28.31 | 47.95 | 10.81 | 26.85 | 45.84 | 11.01 | 23.86 | 39.23 | 11.29 | 19.28 | 31.28 | 8.57 | 19.80 | 32.07 | 7.44 | 18.88 | 30.45 | 7.30 | 17.54 | 29.53 | 7.29 |
| Tottori | 26.22 | 47.06 | 8.37 | 23.94 | 43.78 | 6.64 | 23.53 | 37.50 | 8.23 | 19.08 | 30.19 | 6.56 | 19.70 | 33.18 | 6.88 | 18.16 | 32.04 | 5.51 | 17.59 | 28.04 | 6.36 |
| Shimane | 25.00 | 46.76 | 6.77 | 24.27 | 42.91 | 7.67 | 20.96 | 35.77 | 7.00 | 17.27 | 29.34 | 5.39 | 19.67 | 32.56 | 6.29 | 17.95 | 30.42 | 5.02 | 15.83 | 27.06 | 4.24 |
| Okayama | 27.90 | 48.66 | 9.46 | 26.28 | 44.80 | 9.58 | 24.30 | 40.17 | 10.09 | 19.57 | 32.77 | 7.67 | 19.65 | 33.38 | 7.77 | 18.79 | 30.69 | 7.60 | 17.51 | 28.24 | 7.28 |
| Hiroshima | 27.84 | 46.93 | 11.10 | 25.24 | 42.66 | 9.72 | 24.98 | 40.00 | 11.28 | 19.52 | 32.73 | 7.57 | 20.51 | 33.56 | 8.97 | 18.13 | 30.10 | 7.26 | 17.43 | 28.61 | 7.25 |
| Yamaguchi | 27.52 | 47.27 | 11.25 | 24.85 | 42.41 | 9.78 | 23.16 | 38.94 | 9.86 | 18.47 | 30.54 | 8.06 | 19.79 | 31.79 | 8.14 | 19.09 | 31.84 | 8.26 | 16.67 | 27.92 | 6.88 |
| Tokushima | 27.09 | 47.49 | 9.76 | 24.80 | 41.64 | 9.20 | 24.25 | 40.14 | 9.85 | 18.43 | 31.27 | 7.60 | 18.03 | 29.93 | 6.13 | 17.40 | 28.21 | 6.39 | 16.96 | 26.77 | 6.67 |
| Kagawa | 28.29 | 48.94 | 9.52 | 26.91 | 45.75 | 9.98 | 23.78 | 39.40 | 9.27 | 20.18 | 34.24 | 8.50 | 19.39 | 31.59 | 7.88 | 17.36 | 28.34 | 6.72 | 16.62 | 27.48 | 6.05 |
| Ehime | 26.23 | 46.07 | 9.40 | 24.76 | 42.75 | 9.54 | 22.36 | 38.57 | 9.84 | 18.86 | 31.09 | 7.69 | 18.23 | 30.75 | 7.67 | 18.04 | 30.17 | 6.51 | 15.83 | 26.53 | 6.69 |
| Kochi | 28.44 | 47.64 | 11.80 | 26.69 | 42.01 | 11.01 | 24.18 | 39.29 | 10.64 | 19.90 | 32.01 | 8.90 | 21.94 | 35.42 | 10.41 | 19.29 | 31.60 | 7.81 | 18.21 | 28.03 | 8.14 |
| Fukuoka | 30.01 | 49.97 | 13.38 | 29.56 | 48.20 | 13.50 | 25.35 | 40.27 | 12.58 | 22.70 | 35.09 | 11.78 | 23.60 | 37.71 | 11.90 | 20.37 | 33.30 | 9.58 | 19.84 | 31.78 | 9.27 |
| Saga | 28.46 | 49.83 | 10.14 | 27.87 | 47.96 | 10.50 | 25.30 | 41.86 | 10.64 | 21.30 | 34.68 | 7.71 | 23.09 | 39.60 | 10.39 | 21.66 | 37.46 | 7.41 | 21.21 | 35.84 | 7.51 |
| Nagasaki | 28.00 | 48.46 | 10.56 | 26.63 | 45.45 | 10.28 | 22.47 | 38.31 | 9.56 | 20.61 | 34.90 | 8.86 | 22.29 | 36.90 | 9.43 | 18.85 | 31.91 | 7.47 | 18.93 | 31.68 | 7.76 |
| Kumamoto | 27.24 | 46.52 | 10.70 | 26.71 | 44.29 | 11.75 | 23.60 | 40.25 | 9.53 | 19.66 | 32.76 | 8.59 | 20.88 | 35.50 | 8.16 | NA ^a^ | NA ^a^ | NA ^a^ | 18.43 | 30.81 | 7.70 |
| Oita | 26.44 | 45.43 | 9.94 | 26.82 | 45.24 | 11.58 | 23.28 | 38.52 | 9.98 | 19.72 | 31.99 | 7.65 | 21.71 | 35.42 | 9.26 | 19.14 | 32.39 | 7.02 | 17.77 | 30.73 | 7.05 |
| Miyazaki | 27.54 | 47.77 | 10.43 | 26.46 | 44.80 | 11.06 | 22.78 | 39.41 | 9.11 | 21.07 | 35.10 | 8.41 | 21.27 | 35.57 | 9.03 | 20.05 | 32.75 | 9.33 | 19.19 | 30.53 | 7.80 |
| Kagoshima | 24.54 | 43.77 | 8.48 | 23.90 | 42.58 | 7.80 | 21.55 | 37.50 | 7.89 | 18.38 | 32.26 | 6.82 | 19.74 | 33.50 | 7.98 | 17.42 | 31.22 | 5.50 | 19.06 | 31.21 | 7.94 |
| Okinawa | 27.46 | 43.51 | 11.76 | 25.53 | 40.76 | 11.68 | 23.24 | 37.37 | 10.52 | 20.40 | 32.15 | 9.34 | 20.60 | 31.99 | 9.50 | 18.24 | 29.64 | 7.59 | 19.47 | 29.53 | 8.84 |
