# Supplementary material for: Predicting Smoking Prevalence in Japan Using Search Volumes in an Internet Search Engine: Infodemiology Study
Source: J Med Internet Res. 2022 Dec 14;24(12):e42619. doi: 10.2196/42619 (PMC9798260; doi:10.2196/42619)
Supplement: Multimedia Appendix 2 [file jmir_v24i12e42619_app2.docx]

Table S1. Generalized linear mixed model for both sexes with smoking prevalence as outcome and the search volumes for tobacco related queries as predictors

|  |  | Model 0^a^ | | Model 1 ^a^ | | Model 2 ^a^ | | Model 3 ^a^ | |
| --- | --- | --- | --- | --- | --- | --- | --- | --- | --- |
|  | | B^b^ | P value | B^b^ | P value | B^b^ | P value | B^b^ | P value |
| Total | |  |  |  |  |  |  |  |  |
|  | (Intercept) | 861.15 | P<.001 | -167.99 | P=.79 | 127.64 | P=.74 | -109.19 | P=.89 |
|  | "glo" |  |  |  |  |  |  | -2.13 | P=.17 |
|  | "iqos" |  |  |  |  |  |  | 0.26 | P=.78 |
|  | "vape" |  |  |  |  |  |  | 0.53 | P=.45 |
|  | "iqos" (アイコス) |  |  |  |  |  |  | 0.74 | P=.74 |
|  | "glo”(グロー) |  |  |  |  |  |  | 1.23 | P=.28 |
|  | "cigar" (シガー) |  |  | -0.28 | P=.37 |  |  | -0.31 | P=.40 |
|  | "cigarette"  (シガレット) |  |  | -0.07 | P=.80 |  |  | -0.04 | P=.91 |
|  | "tobacco" (タバコ) |  |  | 0.03 | P=.93 |  |  | -1.02 | P=.36 |
|  | "tobacco" (たばこ) |  |  | -0.34 | P=.61 |  |  | -0.38 | P=.64 |
|  | "ploom tech" (プルームテック) |  |  |  |  |  |  | 0.19 | P=.92 |
|  | "tobacco" (煙草) |  |  | -0.18 | P=.68 |  |  | -0.12 | P=.83 |
|  | "Heat-not-burn tobacco (加熱式タバコ) |  |  | -0.55 | P=.59 | -0.54 | P=.048 | -2.14 | P=.16 |
|  | "Heat-not-burn tobacco" (加熱式たばこ) |  |  | -0.64 | P=.31 |  |  | -0.27 | P=.75 |
|  | "smoking" (喫煙) |  |  | 0.17 | P=.68 |  |  | 0.16 | P=.73 |
|  | " quitting smoking" (禁煙) |  |  | 0.40 | P=.43 |  |  | 1.58 | P=.11 |
|  | "Electronic cigarette" (電子タバコ) |  |  | 0.25 | P=.81 |  |  | 0.36 | P=.78 |
|  | "Electronic cigarette" (電子たばこ) |  |  | 0.01 | P=.98 |  |  | -0.07 | P=.89 |
|  | "cigar" (葉巻) |  |  | 0.34 | P=.37 |  |  | 0.18 | P=.68 |
|  | Data year | -0.42 | P<.001 | 0.09 | P=.77 | -0.05 | P=.78 | 0.06 | P=.87 |
|  |  | AICC^c^ | 324.65 | AICC^c^ | 323.13 | AICC^c^ | 321.53 | AICC^c^ | 308.04 |
|  |  | BIC^d^ | 329.53 | BIC^d^ | 327.71 | BIC^d^ | 326.40 | BIC^d^ | 312.45 |

^a^ Model 0 includes only the survey year and intercept; Model 1 includes search queries excluding tobacco related product names; Model 2 includes search queries selected by the backward selection method, and Model 3 includes all search queries as predictor variables.

^b^ B refers to regression coefficients.

^c^ AICC refers to corrected Akaike’s information criterion corrected for small sample size.

^d^ BIC refers to Bayesian information criterion.

Table S2. Generalized linear mixed model for men with smoking prevalence as outcome and the search volumes for tobacco related queries as predictors

|  |  | Model 0^a^ | | Model 1 ^a^ | | Model 2 ^a^ | | Model 3 ^a^ | |
| --- | --- | --- | --- | --- | --- | --- | --- | --- | --- |
|  | | B^b^ | P value | B^b^ | P value | B^b^ | P value | B^b^ | P value |
| Men | |  |  |  |  |  |  |  |  |
|  | (Intercept) | 1496.15 | P<.001 | 396.69 | P=.63 | 1020.80 | P=.12 | 932.18 | P=.35 |
|  | "glo" |  |  |  |  | -3.42 | P=.009 | -4.19 | P=.04 |
|  | "iqos" |  |  |  |  | 1.02 | P=.05 | 1.17 | P=.30 |
|  | "vape" |  |  |  |  |  |  | 0.16 | P=.85 |
|  | "iqos" (アイコス) |  |  |  |  |  |  | 0.09 | P=.98 |
|  | "glo”(グロー) |  |  |  |  | 2.99 | P=.002 | 3.30 | P=.02 |
|  | "cigar" (シガー) |  |  | -0.55 | P=.16 | -0.59 | P=.08 | -0.50 | P=.26 |
|  | "cigarette"  (シガレット) |  |  | -0.13 | P=.67 |  |  | -0.12 | P=.75 |
|  | "tobacco" (タバコ) |  |  | -0.24 | P=.52 | -2.11 | P=.02 | -2.51 | P=.07 |
|  | "tobacco" (たばこ) |  |  | -0.32 | P=.69 |  |  | -0.32 | P=.75 |
|  | "ploom tech" (プルームテック) |  |  |  |  |  |  | 0.10 | P=.97 |
|  | "tobacco" (煙草) |  |  | -0.53 | P=.29 |  |  | -0.18 | P=.78 |
|  | "Heat-not-burn tobacco (加熱式タバコ) |  |  | 0.55 | P=.62 | -1.86 | P=.03 | -1.91 | P=.27 |
|  | "Heat-not-burn tobacco" (加熱式たばこ) |  |  | -0.74 | P=.33 |  |  | -0.37 | P=.72 |
|  | "smoking" (喫煙) |  |  | 0.81 | P=.17 |  |  | 0.76 | P=.25 |
|  | "quitting smoking"  (禁煙) |  |  | 0.91 | P=.20 | 2.68 | P=.01 | 3.62 | P=.01 |
|  | "Electronic cigarette" (電子タバコ) |  |  | -1.13 | P=.33 |  |  | -0.18 | P=.90 |
|  | "Electronic cigarette" (電子たばこ) |  |  | 0.10 | P=.82 |  |  | -0.35 | P=.57 |
|  | "cigar" (葉巻) |  |  | 0.38 | P=.36 |  |  | -0.09 | P=.85 |
|  | Data year | -0.73 | P<.001 | -0.18 | P=.65 | -0.49 | P=.13 | -0.45 | P=.37 |
|  |  | AICC^c^ | 376.58 | AICC^c^ | 361.09 | AICC^c^ | 356.53 | AICC^c^ | 338.66 |
|  |  | BIC^d^ | 381.46 | BIC^d^ | 365.67 | BIC^d^ | 361.24 | BIC^d^ | 343.07 |

^a^ Model 0 includes only the survey year and intercept, Model 1 includes search queries excluding tobacco product names, Model 2 includes search queries selected by the backward selection method, and Model 3 includes all search queries as predictor variables.

^b^ B refers to regression coefficients.

^c^ AICC refers to corrected Akaike’s information criterion corrected for small sample size.

^d^ BIC refers to Bayesian information criterion.

Table S3. Generalized linear mixed model for women with smoking prevalence as outcome and the search volumes for tobacco related queries as predictors

|  |  | Model 0^a^ | | Model 1 ^a^ | | Model 2 ^a^ | | Model 3 ^a^ | |
| --- | --- | --- | --- | --- | --- | --- | --- | --- | --- |
|  | | B^b^ | P value | B^b^ | P value | B^b^ | P value | B^b^ | P value |
| Women | |  |  |  |  |  |  |  |  |
|  | (Intercept) | 288.91 | P=.002 | -724.76 | P=.18 | -534.79 | P=.12 | -603.99 | P=.34 |
|  | "glo" |  |  |  |  | -0.45 | P=.15 | -1.54 | P=.25 |
|  | "iqos" |  |  |  |  |  |  | -0.41 | P=.63 |
|  | "vape" |  |  |  |  |  |  | 0.47 | P=.45 |
|  | "iqos" (アイコス) |  |  |  |  |  |  | 2.22 | P=.25 |
|  | "glo”(グロー) |  |  |  |  |  |  | 0.14 | P=.88 |
|  | "cigar" (シガー) |  |  | 0.05 | P=.86 |  |  | 0.10 | P=.76 |
|  | "cigarette"  (シガレット) |  |  | 0.36 | P=.15 | 0.36 | P=.08 | 0.44 | P=.14 |
|  | "tobacco" (タバコ) |  |  | -0.15 | P=.53 |  |  | -0.90 | P=.35 |
|  | "tobacco" (たばこ) |  |  | 0.05 | P=.93 |  |  | -0.14 | P=.84 |
|  | "ploom tech" (プルームテック) |  |  |  |  |  |  | -1.19 | P=.44 |
|  | "tobacco" (煙草) |  |  | -0.12 | P=.76 |  |  | -0.16 | P=.74 |
|  | "Heat-not-burn tobacco (加熱式タバコ) |  |  | -1.49 | P=.11 | -1.23 | P=.01 | -1.61 | P=.22 |
|  | "Heat-not-burn tobacco" (加熱式たばこ) |  |  | -0.24 | P=.64 |  |  | 0.20 | P=.77 |
|  | "smoking" (喫煙) |  |  | 0.06 | P=.89 |  |  | 0.14 | P=.77 |
|  | "quitting smoking" (禁煙) |  |  | -0.13 | P=.79 |  |  | 0.68 | P=.47 |
|  | "Electronic cigarette" (電子タバコ) |  |  | 1.09 | P=.26 | 1.11 | P=.11 | 1.02 | P=.40 |
|  | "Electronic cigarette" (電子たばこ) |  |  | 0.05 | P=.85 |  |  | -0.04 | P=.90 |
|  | "cigar" (葉巻) |  |  | 0.22 | P=.42 |  |  | 0.24 | P=.46 |
|  | Data year | -0.14 | P=.003 | 0.36 | P=.17 | 0.27 | P=.11 | 0.30 | P=.33 |
|  |  | AICC^c^ | 318.38 | AICC^c^ | 314.27 | AICC^c^ | 310.89 | AICC^c^ | 302.23 |
|  |  | BIC^d^ | 323.27 | BIC^d^ | 318.85 | BIC^d^ | 315.68 | BIC^d^ | 306.64 |

^a^ Model 0 includes only the survey year and intercept; Model 1 includes search queries excluding tobacco product names; Model 2 includes search queries selected by the backward selection method, and Model 3 includes all search queries as predictor variables.

^b^ B refers to regression coefficients.

^c^ AICC refers to corrected Akaike’s information criterion corrected for small sample size.

^d^ BIC refers to Bayesian information criterion.
